# Assessment of psychosocial aspects in adults in post-COVID-19 condition: the EURONET-SOMA recommendations on core outcome domains for clinical and research use

**DOI:** 10.1186/s12916-025-03927-0

**Published:** 2025-02-11

**Authors:** Stefan Salzmann, Lars de Vroege, Petra Engelmann, Per Fink, Susanne Fischer, Stephan Frisch, Lise Kirstine Gormsen, Katharina Hüfner, Willem J. Kop, Ferenc Köteles, Nadine Lehnen, Bernd Löwe, Christoph Pieh, Victor Pitron, Charlotte Ulrikka Rask, Markku Sainio, Rainer Schaefert, Meike Shedden-Mora, Anne Toussaint, Roland von Känel, Ursula Werneke, Winfried Rief, on behalf of the EURONET-SOMA Group

**Affiliations:** 1 Division of Clinical Psychology and Psychotherapy, Philipps University of Marburg, Gutenbergstraße 18, Marburg, 35032 Germany; 2 Medical Psychology, Health and Medical University Erfurt, Erfurt, Germany; 3 Clinical Centre of Excellence for Body, Mind, and Health, GGz Breburg, Tilburg, The Netherlands; 4 Department Tranzo, Tilburg School of Social and Behavioral Sciences, Tilburg University, Tilburg, The Netherlands; 5 Department of Psychosomatic Medicine and Psychotherapy, University Medical Center Hamburg-Eppendorf, Hamburg, Germany; 6 Clinic for Functional Disorders, Aarhus University Hospital, Aarhus, Denmark; 7 Department of Clinical Medicine, Aarhus University, Aarhus, Denmark; 8 Clinical Psychology and Psychotherapy, Institute of Psychology, University of Zurich, Zurich, Switzerland; 9 School of Medicine, University of St Gallen, St. Gallen, Switzerland; 10 Department of Psychosomatic Medicine and Psychotherapy, Ulm University Medical Center, Ulm, Germany; 11 University Hospital of Psychiatry II, Department of Psychiatry, Psychotherapy, Psychosomatics and Medical Psychology, Innsbruck Medical University, Innsbruck, Austria; 12 Department of Medical and Clinical Psychology, Center of Research On Psychology and Somatic Diseases (CoRPS), Tilburg University, Tilburg, The Netherlands; 13 Department of General Psychology and Methodology, Institute of Psychology, Károli Gáspár University of the Reformed Church in Hungary, Budapest, Hungary; 14 Klinik Und Poliklinik Für Psychosomatische Medizin Und Psychotherapie, Klinikum Rechts Der Isar, Technische Universität München, Munich, Germany; 15 Department for Psychosomatic Medicine and Psychotherapy, University for Continuing Education Krems, Krems, Austria; 16 VIFASOM (Vigilance Fatigue Sommeil Et Santé Publique), Université Paris Cité, Paris, 75004 France; 17 Centre du Sommeil et de la Vigilance-Pathologie professionnelle, APHP, Hôtel-Dieu, Paris, 75004 France; 18 Department of Child and Adolescent Psychiatry, Aarhus University Hospital Psychiatry, Aarhus, Denmark; 19 Outpatient Clinic for Functional Disorders, Helsinki University Hospital, Helsinki, Finland; 20 Department of Psychosomatic Medicine, University and University Hospital Basel, Basel, Switzerland; 21 Institute for Clinical Psychology and Psychotherapy & Department of Psychology, Medical School Hamburg, Hamburg, Germany; 22 Department of Consultation-Liaison Psychiatry and Psychosomatic Medicine, University Hospital Zurich, University of Zurich, Zurich, Switzerland; 23 Department of Clinical Sciences, Division of Psychiatry, Sunderby Research Unit, Umeå University, Umeå, Sweden

**Keywords:** Post-COVID-19 condition, Post-COVID-19 syndrome, Post-COVID-19 condition, Core outcome domains, Instruments, Psychosocial aspects, EURONET-SOMA

## Abstract

**Background:**

Harmonizing core outcome domains allows for pooling data, comparing interventions, and streamlining research evaluation. At the same time clinicians require concise and feasible measures for routine practice. Considering the heterogeneity of post-COVID-19 condition, a biopsychosocial approach requires sufficient coverage of the psychosocial dimension with assessments. Previous recommendations for core outcome sets have serious limitations regarding the psychosocial aspects of post-COVID-19 condition. This paper specifically focuses on psychosocial outcomes for adults with post-COVID-19 condition, providing both a comprehensive set of outcome domains for research and a streamlined clinical core set tailored for routine clinical use.

**Methods:**

In a structured Consensus Development Approach, the European Network to improve diagnostic, treatment, and healthcare for patients with persistent somatic symptoms (EURONET-SOMA) developed psychosocial core outcome domains and assessments regarding post-COVID-19 condition. The experts identified variables and instruments which should be considered in studies on adults suffering from post-COVID-19 condition, and which are feasible in the clinical setting and relevant for research.

**Results:**

We identified three higher-order dimensions with each encompassing several domains: The first higher-order dimension, “outcomes”, encompasses (1) the classification/ diagnostics of post-COVID-19 condition, (2) somatic symptoms (including fatigue), (3) the psychopathological status and mental comorbidities, (4) the physical status and somatic comorbidities, (5) neurocognitive symptoms, and (6) illness consequences. The second higher-order domain “mechanisms” encompasses (7) cognitive components, (8) affective components, (9) behavioral components, (10) social components, and (11) psychobiological bridge markers (e.g., neuroimmunological and psychoneuroendocrinological variables). The third higher-order domain, “risk factors”, includes factors such as (12) socioeconomic status and sociocultural factors, (13) pre-existing mental and somatic health issues, (14) personality factors (e.g., neuroticism), (15) adverse childhood experiences, (16) ongoing disability or pension claim, and (17) social media use. For each domain, specific instruments are suggested for research purposes and clinical use.

**Conclusions:**

The recommended core domains help to increase consistency in a biopsychosocial approach to post-COVID-19 condition across investigations, improve synergies, and facilitate decision-making when comparing different interventional approaches. It allows to better identify relevant subgroups in heterogeneous post-COVID-19 condition populations offering practical tools for routine clinical practice through the clinical core set.

**Supplementary Information:**

The online version contains supplementary material available at 10.1186/s12916-025-03927-0.

## Background

Post-COVID-19 condition or syndrome (often referred to as Long COVID) is the term coined by the World Health Organization (WHO) for the development or continuation of new symptoms 12 weeks after an acute respiratory syndrome coronavirus 2 (SARS-CoV-2) infection, with these symptoms lasting for more than 2 months and not explained by an alternative diagnosis [1]. With 6–10% of SARS-CoV-2-infected individuals reporting long-lasting symptoms, at least 75 million people are affected globally. With a wide variety of more than 200 symptoms reported, post-COVID-19 condition seems an urgent, complex, and massive healthcare problem [2, 3]. Recent studies on post-COVID-19 have identified several prevalent symptoms that persist for up to 2 years after infection: Commonly reported symptoms include fatigue, observed in approximately 28% of cases, and neurocognitive issues such as memory difficulties, dizziness, and brain fog, affecting around 28% of patients. Other symptoms like sleep disturbances (21%), depression (18%), anxiety (13%), and pain (8%) have also been frequently documented among COVID-19 survivors [4]. Given the high variability in these estimates, which reflect diverse populations and study methodologies, we refer readers to recent meta-analyses such as Fernández-de-las-Peñas et al. [4] for a detailed prevalence breakdown of post-COVID-19 symptoms. These symptoms significantly reduce health recovery, everyday functioning, and work capacity 6–12 months post-infection, even in young people with initially mild disease [5]. Since our understanding of and existing treatment options for post-COVID-19 condition are limited, further research and new approaches are necessary [6]. While the onset of the SARS-CoV-2 infection is typically described with a focus on the immunological processes and growing evidence shows the relevance of biological changes, such as persistent alterations in the brainstem of post-COVID-19 patients [7], the chronic course of post-COVID-19 condition requires a broad biopsychosocial perspective that considers the biological (e.g., immunological) and the psychosocial factors that can contribute to or result from the clinical condition. A recent systematic review and meta-analysis has confirmed the relevance of depression and anxiety as co-occurring phenomena and predictive factors of post-COVID-19, but has dramatically shown the scarcity of available evidence on broader psychosocial predictors [8].

This paper specifically focuses on psychosocial aspects of post-COVID-19 in adults and is intended for use by both clinicians and researchers. While the recommendations provide a comprehensive set of outcome domains applicable to both settings, a distinct coreset of measures is highlighted for clinical practice. These core measures (Table 1) are designed to be feasible for routine clinical use, while the broader set of measures is intended to support more detailed research endeavors.

**Table 1:** Clinical core set

Target area	Instrument	Number of items	Clinical cutoff
Somatic symptoms	Somatic Symptom Scale–8 (SSS-8) [27]	8	≥ 9
Anxiety and depression	Patient Health Questionnaire-4 (PHQ-4) [52]	4 (2 for anxiety, 2 for depression)	≥ 3
Health anxiety	Whiteley Index (WI-7)	7	≥ 5
Treatment expectations, previous treatment experiences, and current treatment effects	Generic rating scale for previous treatment Experiences, treatment Expectations, and treatment Effects (G-EEE) [94]	9 (3 for treatment expectations, 3 for previous treatment experiences, 3 for current treatment effects)	-
Cognitive, affective, and behavioral burden of the experienced symptoms	Somatic Symptom Disorder – B criteria Scale (SSD-12) [101]	12	≥ 23
Illness-related disability	(Original) Pain Disability Index (PDI) [78] PDI (adapted version) [149]	7	-

*Notes:* All questionnaires in Table 1 are publicly available and can also be found in the supplementary material S2

The heterogeneity of instruments and outcomes in post-COVID-19 studies limits the accumulation of evidence and its translation into clinical practice. The need for developing a Core Outcome Set (COS) for post-COVID-19 condition to improve data quality, harmonization, and comparability between different studies has been expressed [9]. While available recommendations provide some orientation regarding relevant outcome domains [10], they do not consider various relevant psychosocial aspects sufficiently, despite their relevant role as predictors of post-COVID-19 condition course and as a comorbid condition (i.e., depression). There is evidence that psychological mechanisms play a crucial part in post-COVID-19 condition [6, 11, 12], which is similar to other chronic diseases such as cancer, cardiovascular conditions, or chronic pain conditions (e.g., 7). Moreover, existing conceptual approaches and frameworks argue for the relevance of psychosocial factors in the development and persistence of somatic symptoms in general [13–15]. It is increasingly evident that monocausal associations between one selective pathophysiology and symptoms cannot sufficiently explain chronic conditions such as post-COVID-19 condition; therefore, multidimensional approaches including psychosocial aspects are necessary to provide better clinical services and avoid stigma [6, 11].

To deal with chronic and systemic disease conditions such as post-COVID-19 condition, an interdisciplinary approach is necessary, bringing together the progress of biomedical research in identifying and understanding pathophysiological changes in post-COVID-19 condition [2, 16] with thorough psychosocial evaluation, enabling a holistic explanatory model and leading to multimodal treatment approaches [6, 17]. One reason for not sufficiently considering psychosocial aspects in post-COVID-19 condition may be that researchers and clinicians are unaware of their importance or uncertain about how to assess and integrate psychosocial aspects in clinical practice. Therefore, recommendations for standardized psychological ascertainments are requested that also optimize communication between experts and with patients.

The European Network to improve diagnostic, treatment, and healthcare for patients with persistent somatic symptoms (EURONET-SOMA) is devoted to a multifactorial understanding of persistent somatic symptoms across medicine [18]. Within EURONET-SOMA, we aimed to find a consensus for core domains and instruments to be assessed in post-COVID-19 condition. Focusing on the psychosocial aspects of a multifactorial understanding of post-COVID-19 condition, this paper aims to (i) provide recommendations on which corresponding psychosocial outcome domains and instruments researchers should consider besides biological variables when researching post-COVID-19 condition and (ii) provide clinicians with recommendations for psychosocial assessment tools in clinical practice. This article aims to guide clinicians and inspire further research that helps to better understand and characterize different subgroups of this diverse population of patients suffering from post-COVID-19 condition. There are notable differences in the symptom complexes, and differences of symptom course, comorbid conditions, and finally consequences for disability. For instance, some subgroups may predominantly experience fatigue, sensory or cognitive deficits, others may struggle with additional psychopathological issues like anxiety and depression, while others report the post-COVID-19 condition symptoms to be embedded in a broad spectrum of other somatic symptoms. Defining and recognizing these different clusters is crucial for predicting disease progression more accurately, selecting personalized interventions and allocating treatment resources effectively.

The EURONET-SOMA group has previously provided recommendations on core outcome domains and appropriate instruments for a comprehensive assessment of patients with persistent somatic symptoms [19], and this expertise is used to follow a similar strategy for psychosocial factors in post-COVID-19 condition studies. By this paper, this international group provides recommendations which should help to improve further interdisciplinary research and clinical practice and raise awareness for the importance of the psychosocial aspects of post-COVID-19 condition, in addition to the biological aspects addressed elsewhere (references are provided below). Given that SARS-CoV-2 is not the only virus causing post-acute sequelae, and due to overlapping symptoms with other illnesses and post-viral syndromes, the recommendations given may also apply to other conditions. However, this paper focuses specifically on post-COVID-19 condition.

## Methods

The EURONET-SOMA network used a structured Consensus Development Approach to establish core outcome domains for psychosocial research on post-COVID-19 in adults. Since its founding in 2016, EURONET-SOMA has promoted improved terminology, published recommendations on core outcome domains for clinical trials, and proposed frameworks for understanding persistent physical symptoms. Network members contribute expertise across fields and have led studies on epidemiology, symptom perception, risk factors, and stigma, positioning EURONET-SOMA as a leading network in this area.

An interest group within EURONET-SOMA conducted three iterative discussion rounds, refining suggested domains and instruments through interdisciplinary expertise. This process culminated in in-person meetings in Budapest (2023) and Aarhus (2024), where a final consensus was reached. Although not a formal Delphi process, this approach allowed for iterative discussions and feedback from specialists across clinical psychology, psychiatry, neurology, psychosomatic medicine, and primary care, with substantial expertise in psychosomatic aspects (including neurological aspects) of post-COVID-19. Despite the group’s extensive expertise in a biopsychosocial understanding of diseases and persistent somatic symptoms, we recommend that if screening indicates specific issues, such as neurological concerns, patients should be referred to a specialist in neurology for further assessment.

Our recommendations are intended as screening tools to help clinicians identify whether further specialized assessment is needed based on the identified psychosocial domains. This Consensus Development Approach provided a practical and timely framework to develop standardized assessment recommendations in a field that continues to evolve rapidly. The instruments suggested in this paper are intended to quantify symptom burden rather than verify the etiology of symptoms. This approach aims to capture the impact of persistent post-COVID-19 symptoms on patients’ daily functioning without implying a primary mental health diagnosis. By focusing on the extent and severity of symptoms, we seek to provide a comprehensive assessment while avoiding any stigmatization of these symptoms as purely psychological.

**Fig. 1:**
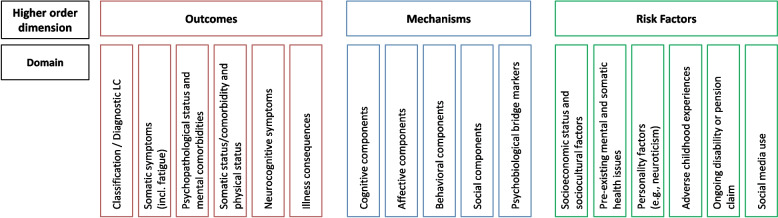
Higher order dimensions and domains which should be considered in research and clinical studies on post-COVID-19 condition

The group’s suggestions were organized hierarchically (Fig. 1): clustered into higher-order dimensions (e.g., outcomes), each encompassing several domains (e.g., somatic symptoms, psychopathological status, mental comorbidities). Each domain can include several subdomains (e.g., somatic symptoms include fatigue and other somatic symptoms), while each subdomain was further specified and complemented with exemplary suggestions for instruments. Criteria for including outcome domains and instruments in the paper were as follows: (i) a clear focus on psychosocial variables that are already recognized as relevant factors in post-COVID-19 condition, or that are well-founded variables in a biopsychosocial understanding of chronic medical conditions in general and (ii) we integrated recommendations on psychobiological features with special relevance for a general biopsychosocial approach (bridge systems), while we leave the specific biomedical aspects of post-COVID-19 condition to the corresponding expert groups (e.g., cardiovascular or pulmonary aspects). The goal was to recommend psychosocial and psychobiological core domains and instruments for post-COVID-19 condition research and provide brief, feasible examples for clinical practice (core set).

The selection of outcome domains and measures was shaped by both current research on post-COVID-19 and the interdisciplinary expertise of the consensus group. The diverse professional backgrounds within the group enabled a comprehensive approach to identifying key domains that are clinically relevant and responsive to the complexities of post-COVID-19 symptoms. The recommended core set of measures is intended for routine clinical application, providing clinicians with feasible tools for screening and assessment (please see Table 1, “Clinical Core Set” for suggestions). For research purposes, however, a more expansive set of tools is included, allowing for in-depth exploration of the various psychosocial dimensions of post-COVID-19.

## Results

The EURONET-SOMA recommendations regarding the outcome domains and the respective instruments for clinical studies on post-COVID-19 condition can be divided into four higher-order dimensions: (1) (clinical) outcomes, relevant for individuals already suffering from symptoms and disorders; (2) mechanisms, which may contribute to the persistence and course of these symptoms and disorders after the SARS-CoV-2 infection; (3) risk factors considering their significant role as predictors, vulnerability aspects, and additional risk factors, which could have existed before the SARS-CoV-2 infection, with relevance for post-COVID-19 condition. Each higher-order dimension encompasses several domains, which may include several subdomains. These subdomains are further explained by specifications and suggestions for instruments assessing the respective subdomain.

The first higher-order dimension, *outcomes*, encompasses (1) the classification/ diagnostic of post-COVID-19 condition, (2) somatic symptoms (including fatigue), (3) the psychopathological status and mental comorbidities, (4) the physical status and somatic comorbidities, (5) neurocognitive symptoms, and (6) illness consequences. The second higher-order dimension, *mechanisms*, encompasses (7) cognitive components, (8) affective components, (9) behavioral components, (10) social components, and (11) psychobiological bridge markers. The third higher-order domain, *risk factors*, includes the factors (12) socioeconomic status and sociocultural factors, (13) pre-existing mental and somatic health issues, (14) personality factors (e.g., neuroticism), (15) adverse childhood experiences, (16) ongoing disability or pension claim, and (17) social media use (Fig. 1).

In the following paragraphs, we describe examples and a selection of instruments, while in the supplementary material (Additional File 1: Table S1), the reader can find a more detailed list of subdomains and applicable assessment tools. We will first describe the domains and instruments for research purposes. Of note, the recommendations for instruments should be adapted according to the specific focus of a clinical study or the clinical use, e.g., if clinicians wish to assess the psychosocial aspects in post-COVID-19 condition, they may refer to recommendations for brief and feasible assessments (core set, 5–6 instruments, Table 1) for clinical use in daily routine care.

### (Clinical) outcomes

#### Classification/diagnostics of post-COVID-19 condition

For the classification and diagnostics of post-COVID-19 condition, it seems crucial to assess whether individuals had a laboratory-confirmed infection (i.e., SARS-Cov-2-IgG antibody test), if there has been an expert evaluation assessing a SARS-CoV-2 infection as clinically probable, or whether the assumption is based on the patient’s belief that she/he has been infected. Previous studies have shown that the belief in having been infected was associated with more persistent somatic symptoms than having a laboratory-confirmed infection [20]. Since there are several definitions of post-COVID-19 condition/ Long COVID as well as several infection waves and virus variants, it is helpful to assess the date of infection, symptom duration, and whether individuals suffer from ongoing or newly developed symptoms after the acute SARS-CoV-2 infection. It is also important to consider factors such as home testing and known exposure to infected individuals, especially since many patients did not have access to formal testing earlier in the pandemic. Accumulating research also indicates that the post-vac syndrome can also produce post-COVID-19 condition-like symptoms in at least some individuals (19); thus, the amount, timing, and the kind of vaccine may also be relevant.

#### (Persistent) somatic symptoms (incl. fatigue)

Persistent somatic symptoms are very common following SARS-CoV-2 infection, with a median of 72.5% of individuals reporting at least one ongoing (somatic or mental) symptom [21]. In a comprehensive study comparing COVID-19 patients with matched controls, it was found that 12.7% of patients continued to experience at least one significant symptom 90–150 days after their COVID-19 diagnosis, even after considering pre-existing symptoms and general symptom trends in the population without the infection [22]. However, assessing the prevalence is difficult since there is no consensus about rating the causality of symptoms, e.g., which symptoms can be attributed to COVID-19 [22]. Post-COVID-19 condition is a multi-organ disease [16], and patients suffering from this disease frequently report somatic symptoms across several domains, such as fatigue, pain, shortness of breath, or sleeping problems [5, 23–25].

To gain a clearer picture of the (persistent) *somatic symptom burden* in post-COVID-19 condition, clinical studies should assess the somatic symptom burden, which can be measured with the Patient Health Questionnaire-15 [26]. This questionnaire is widely used and one of the best-validated instruments for measuring the presence and severity of common somatic symptoms. The Somatic Symptom Scale-8 (SSS-8) is an abbreviated reliable, and valid 8-item version of the PHQ-15, assessing gastrointestinal, pain, fatigue, and cardiopulmonary aspects of the general somatic symptom burden; this instrument may be a good alternative if the instrument should be even more feasible, as completion by the patient takes only 1 min [27]. Of note, the SSS-8 uses a 7-day time-frame whereas the PHQ-15 asks about the past 4 weeks. Also important: the scoring (0–3 points: minimal; 4–7: low; 8–11: medium; 12–15: high; 16–32: very high somatic symptom burden) needs only 1 min to enable healthcare professionals to obtain a valid score in everyday clinical practice. Importantly, the PHQ-15 (and related instruments) is suggested here to gauge somatic symptom burden in post-COVID-19 patients, regardless of underlying etiology, with a focus on symptom impact rather than implying a somatoform disorder. Clinical context and further assessments are recommended to interpret PHQ-15 scores accurately and avoid characterizing post-COVID-19 symptoms as primarily mental health-related.

The bodily distress syndrome (BDS) checklist [28] with its 25 items is a screening tool for *functional somatic disorder* [29]. Functional somatic disorder is a relatively new umbrella term suggested by the EURONET-SOMA group for conditions involving persistent and burdensome physical symptoms that cannot be understood as purely somatic or purely mental. If the BDS indicates that a functional somatic disorder is already present or suspected, further diagnostics and treatment may be necessary. The BDS checklist can also be used as a measure of physical symptom burden. Numeric Rating Scales (NRS) as recommended by the EURONET-SOMA group [19] can be used for the efficient assessment of symptom intensity and symptom interference.

Although the PHQ-15 (or the SSS-8) allows for measuring tiredness or fatigue, a more specific instrument for assessing *fatigue* seems appropriate since fatigue is a prevalent and disabling symptom in individuals suffering from post-COVID-19 condition [5]. Fatigue is a cardinal post-COVID-19 condition symptom, the most common symptom 6 to 12 months after acute infection and one of the major causes of substantial interference with daily life in post-COVID-19 condition patients. One established instrument for assessing fatigue is the Chalder Fatigue Scale (CFS), which comprises 11 items [30]. Each item is answered on a 4-point scale from the asymptomatic to maximum symptomology. The CFS covers the severity of physical and mental fatigue, has good psychometric properties, and has been utilized in clinical and non-clinical samples [31, 32] as well as in several studies on post-COVID-19 condition [33, 34], making it valuable tool for accurately and efficiently assessing fatigue in post-COVID-19 condition patients. Fatigue and *post-exertional malaise *(PEM) assessment are also of particular interest due to the overlapping symptoms with myalgic encephalomyelitis/ chronic fatigue syndrome (ME/ CFS) [35]. A questionnaire explicitly assessing PEM is the DePaul Post-Exertional Malaise Questionnaire (DSQ-PEM) [36]; this 5-item questionnaire assesses the frequency and severity of PEM during the last 6 months.

For *shortness of breath*, which is also a common symptom in post-COVID-19 condition patients [5], the questionnaire Dyspnoea-12 (45) can be applied. For pain assessments, we recommend using a Numeric Rating Scale (NRS; see chronic pain chapter in ICD-11 [37]). *Sleeping problems*, an additional common symptom in post-COVID-19 condition individuals, can be assessed with the 19-item Pittsburgh Sleep Quality Index (PSQI) [38] or the more efficient 7-item Insomnia Severity Index (ISI) [39].

#### Psychopathological status and mental comorbidities

Psychological distress such as depression, anxiety, perceived stress, loneliness, and worry are prospectively associated with an increased risk of developing post-COVID-19 condition [40]. Meta-analyses indicate that—besides prevalent somatic symptoms such as fatigue—psychopathological factors such as depression and anxiety are also prevalent in patients 1 year after the acute SARS-CoV-2 infection [41, 42]. In a prospective cohort study, in both infected and non-infected individuals, the best predictor of persistent symptoms was *depressive symptoms* at the pandemic’s start [43]. Depression in general is one of the leading causes of disability worldwide and substantially contributes to the overall disease burden [44]. However, depressive symptoms are not only debilitating but have been shown to predict long-term outcomes such as fatigue [45]. Due to their high prevalence and its importance as a risk factor for other outcomes, depressive symptoms should be assessed. The Patient Health Questionnaire-9 (PHQ-9) [46] is a widely used and well-validated instrument to assess depressive symptomatology. With its 9 items covering crucial aspects of depressive symptoms for the past 2 weeks, including suicidal tendencies, the PHQ-9 is a highly reliable, brief, and time-efficient self-report tool. The 8-item-version (PHQ-8) [47] may be of interest if the assessment of suicidality could cause problems. To accurately assess anxiety and mood disorders in individuals with post-COVID-19 conditions, it is critical to minimize overlap with somatic symptoms that are part of the condition itself. Given the overlap between somatic symptoms of post-COVID-19 and items on traditional mood measures like the PHQ-9, we recommend considering alternative assessments that minimize the influence of somatic symptoms. Measures such as the Hospital Anxiety and Depression Scale (HADS) [48] may provide a more accurate evaluation of affective symptoms in this population. This approach reduces the risk of misattributing physical symptoms to psychopathological conditions and ensures a more precise assessment.

Besides depressive symptoms, *anxiety symptoms* are also prevalent in post-COVID-19 condition patients [49]. The Generalized Anxiety Disorder-7 (GAD-7) questionnaire is a brief and valid instrument to assess the severity of anxiety symptoms [50]; however, there is an ultra-brief scale that assesses depression and anxiety with only four items in total: The Patient Health Questionnaire-4 (PHQ-4) screens for anxiety and depression (GAD-2 and PHQ-2) [51] and is—despite its brevity—still reliable and valid [52]. Another brief option for assessing anxiety and depressive symptoms is the Symptom Checklist-8 (SCL-8) with four items for anxiety (SCL-4anx subscale) and four items for depressive symptoms (SCL-4dep) [53]. A study comparing individuals with an acute SARS-CoV-2 infection to those with post-COVID-19 condition indicated different trajectories of depressive and anxiety symptoms in both groups; this highlights the need for monitoring mental health and adequate treatments of mental health issues in addition to the treatment of physical consequences of post-COVID-19 condition [54].

*Health anxiety* may also play an essential role in patients suffering from post-COVID-19 condition. Research indicates that health anxiety is linked to negative interpretation biases [55], which can exacerbate and prolong somatic symptoms. These processes are particularly relevant in the context of post-COVID-19, where persistent symptoms remain a significant issue. For the assessment of health anxiety, the Whiteley Index-7 (WI-7) is one of the most widely used instruments since it is a valid and efficient questionnaire [56]; there is also a revised and validated 6-item version available [57]. Although post-COVID-19 condition can affect patients in the full range from a very mild acute disease to very severe forms [58], *post-traumatic stress disorder *(PTSD) may be more prevalent in those having experienced a severe acute SARS-CoV-2 infection and treatment in the intensive care unit (ICU) [59]. It may also manifest in individuals with post-COVID-19 condition who witness family or friends with COVID-19-related complications. To assess PTSD symptoms, the PTSD Checklist for DSM-5 (PCL-5) is a psychometrically sound measure of PTSD symptoms [60]; it may be relevant to anchor questionnaire items to COVID-19-related traumatic events.

While the instruments mentioned above allow for assessing the severity of several constructs, such as anxiety or depression, they do not allow for making diagnoses. The gold standard for diagnosing mental disorders is a validated clinical interview such as the structured clinical interview for DSM-5 (SCID-5) [61], the open-access clinician-administered diagnostic interview for mental disorders (DIPS) [62], or more efficient assessment tools such as the Primary Evaluation of Mental Disorders (PRIME-MD) [63] or the Mini International Neuropsychiatric Review (M.I.N.I.) [64]. However, while structured interviews are the gold standard to verify mental disorders in research, they are typically too time-consuming for many clinicians, and in addition they require some special training.

#### Physical status and somatic comorbidities

Although this paper focuses on the psychosocial aspects of post-COVID-19 condition, biomedical factors should be also assessed since a patient’s physical status or existing comorbidities interact with the psychosocial variables, resulting in an individualized patient experience. We refer to other reviews for biomedical findings [2]. For specific organ sections, several recommendations are already available (e.g., with a cardiovascular focus see [65]; for specific somatic questions, see also the guidelines such as the British National Institute for Health and Care Excellence (NICE) COVID-19 guideline [66] or the German Association of the Scientific Medical Societies (AWMF) S1-guideline [67]). Further, we suggest to consider applying the *6 min walk test* [68], *one minute sit to stand test* [69], which can be performed in a limited setting such as general practice, or the *JAMAR® grip strength test* [70] to assess a patient’s physical performance status and disability. While most recommended domains and instruments in this paper refer to or are patient-reported outcomes (PROs), these tests are quick behavioral tests, which can be easily applied. Besides (pre-existing) somatic comorbidities and current medications, previous treatments and the duration and onset of symptoms should be assessed with a structured medical history and hospital charts should be used to note ICD-10/ 11. Instruments such as the Charlson Comorbidity Index (CCI) [71] and/or a self-report measure (for instance, the Self-administered Comorbidity Questionnaire (SCQ) [72]) cover important *medical comorbidities* with prognostic value.

#### Neurocognitive symptoms

Neurocognitive impairments such as *brain fog and dizziness* are also some of the most prevalent and debilitating symptoms of post-COVID-19 condition, and these symptoms can substantially impact work ability [5]. Subjective cognitive complaints can interfere with activities in daily life and include impairments from the patient’s perspective. Accordingly, the Cognitive Complaints Questionnaire – Participation (CoCo-P) assesses cognitive complaints in daily life across several domains and the impact of these complaints on participation [73]. This questionnaire has a version for patients and one for relatives so that both perspectives can be compared.

In contrast to subjective cognitive complaints, objective cognitive performance should always complement the diagnosis of neurocognitive deficits and can be measured by valid tests. For neurocognitive functioning such as *attention deficits*, neuropsychological test batteries such as the Test battery of Attentional Performance (TAP) [74] may be applied. The recommended instruments serve as preliminary screening tools, providing an initial indication of possible issues in areas such as cognitive function. For instance, the included attention test is one example and does not cover the entire range of cognitive abilities. If screenings indicate potential concerns, further in-depth assessments by specialists are recommended to evaluate specific cognitive domains, such as processing speed, memory, or attention, in greater detail. Readers with a special interest in this field are referred to more specific sources [67]. Cognitive difficulties may be influenced by psychosocial factors such as anxiety, low mood (discussed later in this paper), or pre-existing conditions like attention-deficit hyperactivity disorder (ADHD). While these factors contribute to a range of possible influences and do not imply that these symptoms are indicative of functional disorders, they should be considered as one potential aspect in a comprehensive assessment.

#### Illness consequences

One of the most relevant generic patient-reported outcomes is health-related *Quality of Life *(QoL), which should encompass psychological and physical functioning. Post-COVID-19 condition is associated with impaired QoL and functioning due to the manifold persistent symptoms [75]. The Short Form Health Survey (SF) 12 [76] is the abbreviated version of the SF-36 and is one of the most frequently used assessment tools for QoL. The European Quality of Life 5 Dimensions 5 Level Version (EQ-5D-5L) is an even shorter standard questionnaire for QoL assessment [77]. In some studies, this questionnaire has also been used to assess QoL in post-COVID-19 condition patients (61). Besides QoL, another essential outcome that needs to be assessed in clinical studies on post-COVID-19 condition is *patients’ disability*: For instance, the adapted version of the Pain Disability Index (PDI) [78] assesses patients’ illness-related disability in seven domains of daily living. Another valid option to measure patients’ disability is the World Health Organization Disability Assessment Schedule 2.0 (WHODAS 2.0) [79]. A very brief option to assess patients’ disease-specific disability is the one-item post-COVID Functional Status Scale (PCFS) [80]. Of note, disability in post-COVID-19 patients may be multifactorial, arising from both the direct effects of COVID-19 and, for some patients, the prolonged impairments associated with ICU stays. Research shows that ICU survivors frequently experience lasting disabilities, which contribute to increased healthcare costs and lower quality of life [81]. *Healthcare costs* and healthcare utilization are also highly relevant outcomes. Similar to patients with persistent somatic symptoms, patients with post-COVID-19 condition cause high costs in the healthcare system due to the large number of different symptoms and the high number of doctor visits to different specialists [82]. Healthcare cost reduction is an important objective outcome that informs the decisions of healthcare insurances and governments. *Healthcare utilization* can be assessed using the Schedules for Clinical Assessment in Neuropsychiatry (SCAN) [83] or more specific instruments.

### Mechanisms

Assessing the psychosocial aspects in post-COVID-19 condition should also incorporate mechanisms and processes of change as important mediators and outcome predictors. Mechanisms are not exclusively linked to specific syndromes (like clinically relevant symptoms), but they can be observed in patients with various medical severities, and even in healthy individuals. Identifying these modulators of the disease course may help influence chronic illness courses of post-COVID-19 condition, in particular since many of these psychological mechanisms (of post-COVID-19 condition) are modifiable factors. In the following paragraphs, we describe the domains of cognitive, affective, behavioral, and social components, as well as psychobiological bridge markers.

#### Cognitive components

Patients’ *expectations*, as one of the primary mechanisms underlying placebo and nocebo effects [84], are a crucial predictor of health outcomes and substantially influence treatment success in medical and psychological interventions across several diseases [85, 86]. *Illness beliefs*, defined as patients’ perceptions and assumptions about the symptoms, such as the causes, the timeline, control, and consequences of one’s illness [87], have also been shown to be relevant predictors of health [88, 89]. The role of illness beliefs and expectations in post-COVID-19 condition is also indicated by studies showing that the belief in having been infected was associated with more persistent somatic symptoms after COVID than having a laboratory-confirmed infection [12, 20]. A belief about the expected symptom severity in case of a SARS-CoV-2 infection predicted experiencing COVID-like symptoms weeks later [90]. Of note, a recent meta-analysis indicated that many experienced side effects of COVID vaccines also emerged in the placebo arms of vaccination studies [91], arguing for an essential role of psychological functions and expectations in vaccination side effects (and potentially the post-vaccination syndrome). This does not suggest that symptoms are imaginary, it simply suggests that mechanisms outside the direct immunological trajectories of the vaccine are also important. In addition to the patient’s own illness beliefs, the perceptions and experiences of family and friends can also be impactful. For instance, the illness beliefs of close social contacts, and whether family members or friends have experienced long COVID themselves, may shape the patient’s understanding of their condition and influence their emotional and psychological response. These social dynamics warrant further exploration and consideration in both clinical assessments and research on post-COVID-19 outcomes.

The Treatment Expectation Questionnaire (TEX-Q) is a valid, generic multidimensional scale measuring patients’ expectations regarding medical and psychological treatments [92], and compares different expectation constructs (i.e., expected treatment benefit and negative impact of the treatment) [93]. An even more efficient instrument is the Generic rating scale for previous treatment Experiences, treatment Expectations, and treatment Effects (G-EEE); this very compact generic screening instrument aims to assess patients’ positive and negative treatment expectations, but also the effects on general clinical outcomes while assessing both prior treatment and current treatment experiences [94]. Regarding the assessment of illness beliefs, the Brief Illness Perception Questionnaire (B-IPQ) [95] is a widely used 9-item questionnaire with decent psychometric properties indicated by meta-analytic findings [88].

Other important predictors of symptom persistence and mediators of treatment outcome are *fear avoidance beliefs* (e.g., “I am afraid that I will make my symptoms worse if I exercise”) [96, 97]. Fear avoidance beliefs can be assessed using the Cognitive and Behavioral Responses to Symptoms Questionnaire (CBRQ), which is a reliable and valid measure; the CBRQ-SF is the short version of the questionnaire and is recommended due to its more robust factor structure and brevity [98]. Other relevant factors for experiencing somatic symptoms are interoception and somatosensory amplification: Dysfunctional interoception has been recognized as a crucial factor in several disorders with accompanying somatic symptoms [99], while *somatosensory amplification* refers to the vicious cycle of illness anxieties, increased attention to symptoms, the *amplified intero-/perception and catastrophizing of symptoms* and can be measured using the Somatosensory Amplification Scale (SSAS) [100].

The Somatic Symptom Disorder – B criteria Scale (SSD-12) is a reliable and valid self-report instrument measuring the cognitive, affective, and behavioral aspects of DSM-5 *Somatic Symptom Disorder* [101, 102]. This brief 12-item scale measures the psychological burden related to somatic symptoms or associated health concerns. Combining the PHQ-15 or the SSS-8 with the SSD-12 is an easy-to-use, time- and cost-efficient approach to identify individuals at risk for somatic symptom disorder [103]; of note, the C criterion (time) of persistent symptoms needs to be assessed as well.

#### Affective components

Subclinical affective components may play a crucial role as mediators/mechanisms of change since affect has generally been shown to be associated with health outcomes. For instance, there is evidence that the induction of negative affective states leads to elevated somatic symptom reports in functional somatic syndrome patients [104], also highlighting the role of emotion regulation. One widely applied measure is the Positive And Negative Affect Schedule (PANAS) [105] which allows the assessment of *positive and negative affect*. Since this scale comprises 20 items, the brief version with only 10 items might be more appropriate for clinical use [106]. As mentioned above, the SSD-12 also allows for an assessment of the psychological aspects of persistent somatic symptoms, including anxiety and other affective components.

To assess anxiety and mood, we recommend brief screening tools for initial assessment. However, for cases requiring a more comprehensive evaluation, PROMIS (Patient-Reported Outcomes Measurement Information System) [107] offers validated measures that focus on cognitive aspects of anxiety and depression, while also addressing associated physical symptoms. This approach supports a multidimensional understanding of post-COVID-19 symptoms, particularly where cognitive and physical aspects intersect.

#### Behavioral components

*Illness behavior*, which describes how patients cope with their illness and encompasses features such as healthcare use (see above), urging physicians to do investigations, taking medication, work disability, avoiding physical activity, and expressing symptoms, is also a relevant component which should be mentioned here as a behavioral component [108]. There is sound evidence that behavioral components such as *physical activity* are associated with various positive effects such as improved immunological health, managing physical syndromes, reduced pulmonary complications, and improved cardiovascular health [109, 110], although some experts are very cautious in providing physical activity in patients with post-COVID-19 condition [111]. While physical activity can be assessed with questionnaires such as the International Physical Activity Questionnaire (IPAQ) [112], we recommend a more objective assessment with a wearable activity tracker. Measuring daily steps may provide an objective indicator of one’s activity and is an interesting additional outcome in combination with self-report measures. Again, illness behavior can also be assessed with the SSD-12.

#### Social components

Social aspects such as having *social support* are positively associated with more positive health outcomes, while perceived stigma has various deleterious effects on various health variables [113]. The Multidimensional Scale of Perceived Social Support (MSPSS) [114] may be applied to assess social support, while perceived stigma may be rated on a single item. Another option is the Oslo Social Support Scale (OSSS-3) [115], a brief and efficient 3-item self-report measure. *Loneliness* is associated with impaired quality of life and increased all-cause mortality [116, 117], which can either be assessed with questionnaires measuring perceived loneliness (e.g., UCLA Loneliness Scale (ULS) [118]), or as a general sociodemographic question (e.g., “Are you feeling lonely?”) [119].

#### Psychobiological bridge markers

The term “bridge markers,” derived from network analysis concepts of “bridge systems,” refers to symptoms that connect different symptom clusters and facilitate interactions between biological and psychosocial domains. In post-COVID-19, symptoms like fatigue can act as these bridges, linking and influencing multiple symptom networks. Although this paper focuses on psychosocial aspects, *psychoneuroimmunological *and *psychoneuroendocrinological markers* should be considered when researching post-COVID-19 condition since they can be seen as *psychobiological bridge markers* linking mental and biological processes. These markers are also indicators of allostatic load, defined as the cumulative biological burden of chronic stress and previous life events [120]. Markers used to assess allostatic load can have direct effects on psychological aspects: For instance, pro-inflammatory cytokines and C-reactive protein (CRP) released during infections, are known to alter the central nervous system’s neurophysiological processes and cause sickness behavior with depressive-like symptoms, including low mood, decreased drive to act, and attention problems [121–123]. In addition, immunological markers may predict the responsiveness to psychotherapy [124, 125], can moderate the effects of psychological interventions in chronic somatic diseases [126] and anti-depressant medication effects [127], and show interactions with physical and mental symptoms [128]. There is interesting research on identifying biological markers, immunological profiling, or underlying mechanisms in patients suffering from post-COVID-19 condition: In one study, cortisol was the most significant individual predictor of post-COVID-19 condition [129]. Cortisol has repeatedly been associated with fatigue syndromes [130] and predicts responsiveness to psychological therapies [131] and antidepressant treatment [132]. Other studies have discussed the role of inflammation or thrombotic tendency for COVID-19 and post-COVID-19 condition [16, 133]. However, there is no specific biomarker for post-COVID-19 condition yet, and previous findings have to be corroborated by other studies. We refer the interested reader to other resources with a stronger focus on biomedical aspects of post-COVID-19 condition (e.g., 2).

### Risk factors

Besides outcomes as well as mechanisms, the third higher-order dimension incorporates risk factors. Previous studies have indicated that *socioeconomic status and sociocultural factors* such as female *sex* and lower socioeconomic status and sociocultural factors are risk factors for developing post-COVID-19 condition [134]. Therefore, sex, gender, race and ethnicity, education, and socioeconomic status as well as age should be assessed. We suggest assessing both sex (biological aspects, such as genes and hormones) and gender (socially influenced roles and exposures) as factors potentially influencing post-COVID-19 outcomes [135, 136]. Research suggests that these factors often interact with other sociodemographic elements, such as socioeconomic status and educational level, which can influence chronic illness susceptibility. We recognize that the combined effects of these factors may contribute to the risk and severity of post-COVID-19 symptoms, with sex and gender potentially acting as confounding variables in understanding these outcomes. Assessing work (dis-)ability due to post-COVID-19 condition may also be of relevance, since it is a risk factor for the persistence of symptoms [137, 138]. Access to specialty post-COVID-19 services may be influenced by geographic factors, such as rural versus urban residence, which could impact patient outcomes.

### Additional risk factors

In the context of post-COVID-19 condition, recognizing an individual’s vulnerability is essential since the virus does not encounter a blank slate but meets a complex set of pre-existing conditions that significantly shape susceptibility as well as outcomes. The occurrence and progression of post-COVID-19 condition is influenced significantly by this factor, which comprises the accumulated burden from previous somatic diseases and mental disorders. *Previous somatic diseases* that increase the risk of post-COVID-19 condition include obesity, asthma, chronic obstructive pulmonary disease, diabetes, immunosuppression, ischemic heart disease, and previous hospitalization or intensive care unit (ICU) admission [139]. Since also the presence of *previous mental disorders* is a predictor of post-COVID-19 condition [140], assessing the history of mental health issues is critical. *Neuroticism*, as one crucial personality trait, is an individuals’ disposition to experience negative affect and is considered a risk factor for psychopathology [141]; neuroticism is associated with a tendency to interpret ambiguous information as threatening and is linked to an increased sensitivity to negative information, somatic sensations, and stressors [142]. The Big Five Inventory (BFI-10) assesses 5 personality traits (extraversion, neuroticism, openness, conscientiousness, agreeableness) on a highly economic scale with only 10 items in total and two items assessing neuroticism [143]. Stress during childhood can have long-term (negative) effects on an individual’s health, and it predicts mental health conditions such as depression or anxiety, but also cardiovascular hazards [144, 145]. Therefore, assessing *adverse childhood experiences* seems relevant and can be done with the Adverse Childhood Experiences Questionnaire (ACE) [146].

Diagnostics and treatment of post-COVID-19 condition (symptoms) may be further complicated by a variety of socio-psychological factors capable of influencing patient outcomes and the efficacy of therapeutic interventions. One significant additional risk factor may be a patient’s *ongoing disability* and an ongoing process of *pension claims* where legal issues and personal expectations of disability benefits may interact with symptom report and treatment engagement, potentially skewing clinical assessments and outcomes. This is of particular relevance if patients got infected with COVID at the workplace. Ongoing disability in post-COVID-19 patients can be assessed using validated instruments described earlier in the manuscript, including the adapted Pain Disability Index (PDI), which captures functional impairment across various domains. For capturing additional socioeconomic aspects, such as pension claims and health service utilization, we recommend structured questions during patient interviews and health record review, as these approaches can yield valuable insights into the broader socioeconomic and healthcare impacts of post-COVID-19.

Moreover, the widespread *use of social media* as a source of information about post-COVID-19 condition, particularly in specific patient advocacy groups, may also play a dual role: While it offers support and valuable information exchange, it may also propagate critical attitudes toward state policies and medical advice, leading to skepticism and reduced adherence to recommended treatments. This dual influence of social media has been documented in the context of other “health scares,” which are highly publicized health threats that increase public concern [147]. Prospective studies in patients with post-COVID-19 condition suggest that trust in doctors/scientist and government/journalists predict a lower symptom burden, while social media interacted with that trust [148]. In managing post-COVID-19 condition, healthcare providers may benefit from considering these factors as potential influencing factors to effective assessment and treatment, necessitating a more nuanced approach to patient education and engagement in their care process. Altogether, these variables help to subcategorize different subgroups with post-COVID-19 condition and can indicate relevant aspects for different prognosis.

### Clinical core set

Despite the many suggested core domains and instruments to assess these domains, the number of items needs to be adapted to the patient’s capacity and readiness as well as to the clinician’s time constraints which makes highly efficient instruments necessary to be feasible in a clinical setting. Therefore, we suggest the following core set to assess some of the most crucial psychosocial domains in post-COVID-19 condition patients in a highly efficient way (Table 1; all questionnaires can be found in the supplementary material S2).

## Discussion

While studies on post-COVID-19 condition require a broad biopsychosocial perspective, we give recommendations for the psychosocial and psychobiological part, recommending core outcome domains and instruments. This paper further aims to provide clinicians with brief and efficient instruments that are feasible and applicable in daily clinical practice. These recommendations, while not replacing other quality criteria like CONSORT, aim to enhance comparability and insights into post-COVID-19 condition research.

Other recommendations regarding core outcome sets for post-COVID-19 condition include those from the PC-COS study using a Delphi consensus approach [10, 150]. While our recommendations show some overlap, relevant differences to the PC-COS recommendation are of note: we have a strong and comprehensive focus on the psychosocial aspects of post-COVID-19 condition since this part is often neglected by purely biomedical approaches. For instance, the PC-COS recommendations do not specifically mention depression [10], although meta-analytic evidence confirms higher levels of depression and anxiety in individuals with post-COVID-19 condition compared to controls and that both mental health conditions predict the course of post-COVID-19 condition [8, 40, 43, 54]. In this paper, we also want to raise awareness of the importance of evidence-based psychosocial aspects in healthcare professionals when dealing with post-COVID-19 condition patients. It is also built on the experience and understanding of complex biopsychosocial relationships underlying many conditions with persistent somatic symptoms that have been extensively researched by the EURONET-SOMA group in former studies [6].

One strength of this article is that we give recommendations for research purposes; however, with 5–6 instruments recommended for use in clinical practice, a feasible set of brief and efficient instruments is also provided. While the instruments we recommend, such as the PHQ-15, are effective in capturing the burden of somatic symptoms, it is important to interpret these results as measures of symptom impact rather than definitive diagnoses or indicators of etiology. This approach avoids implying that persistent symptoms are primarily mental health-related and ensures a focus on the severity of patients’ functional impairment. Interpreting results within a broader clinical context can help clinicians better understand the effects of post-COVID-19 without risking stigmatization.

Different phenotypes of post-COVID-19 condition suggest varied underlying mechanisms, each with implications for treatment [16]. Identifying patient subgroups based on pathophysiological and psychobiological mechanisms could advance research significantly. Notably, SARS-CoV-2 is not the only virus associated with post-acute sequelae, as symptoms overlap with conditions like ME/CFS [151]. While similar mechanisms may underlie different illnesses, patients with similar complaints may also have distinct mechanisms, highlighting the need for subtyping. The limited understanding of biological mechanisms in other post-infectious syndromes further supports this approach. Symptoms may arise not only from specific biomedical issues but also from how the brain processes sensory information, with top-down influences sometimes outweighing bottom-up sensory input [14]. This framework may help explain the disparity often observed between self-reported symptoms and objective test findings in chronic conditions, including post-COVID-19 [11, 152].

Findings and assumptions that psychosocial factors seem relevant in post-COVID-19 condition [11, 20] emphasize the importance of considering placebo and nocebo mechanisms such as patients’ expectations, positive patient-provider interaction, and previous experiences (learning) for post-COVID-19 condition. Besides patients’ expectations and prior learning experiences, the patient-provider relationship is also a crucial mechanism driving placebo or nocebo effects, may thus influence treatment outcomes significantly and should inform future treatments [84, 85]. Since patients’ expectations and beliefs are crucial for recovery, it is important to challenge the narrative that conditions like chronic fatigue syndromes or post-COVID-19 conditions are incurable. The Oslo Chronic Fatigue Consortium offers a hopeful, research-based perspective, suggesting these conditions result from the brain’s response to various factors rather than being a specific disease [153]. They propose that symptoms persist if perceived as threatening and advocate against prolonged rest and isolation. Instead, they support a gradual return to normal activities and call for open dialog, including recovered patients’ perspectives.

Considering additional risk factors for a more complicated course of post-COVID-19 condition may prove helpful in predicting an individual’s patient trajectory. In this paper, we suggest considering factors such as an ongoing pension claim, or the excessive use of social media from specific patient advocacy resources as relevant aspects that may be easy to assess with a few questions during an interview or in a questionnaire but may provide highly relevant information for diagnostics, treatment, and prediction of trajectories. This additional information may help clinicians adjust their approach to a specific patient’s needs making a personalized approach possible and a better outcome more likely. Therefore, our recommended domains and variables should help evaluate different risk profiles for favorable and unfavorable courses but also indicate special topics for individualized treatment planning. For that, it is also advisable to identify resilience factors and resources in the patient to find a salutogenic path with them. The recommendations given in this paper are not set in stone, but can be adapted to the needs of applicants and latest findings.

The recommended core domains aim to improve synergies of clinical studies and may also facilitate decision-making when comparing different interventional approaches. The recommendations also aim to increase consistency across investigations in post-COVID-19 condition research. However, a common problem is that individuals often seek straightforward explanations and strive for the one biological cause for a debilitating syndrome such as post-COVID-19 condition, whereas complex conditions such as post-COVID-19 condition are typically better understood if the interaction of biological and psychosocial factors is considered and adapted to a personalized approach [11]. Here, the field can learn from successful approaches how to broaden patients’ as well as healthcare professionals’ perspectives in related conditions such as chronic pain [154]. A better understanding of psychosocial factors contributing to post-COVID-19 condition and a more integrative approach may also reduce the perceived stigma of individuals suffering from post-COVID-19 condition.

### Limitations

This paper is a product of the EURONET-SOMA group consisting of international experts in Europe; therefore, this process depended on the persons being involved, and although three discussion rounds were used, it did not follow a more structured approach such as the Delphi process. Different approaches of other papers may stimulate discussions and bring forward new research and better treatments. Further, one important limitation of this paper is the lack of patient involvement. Therefore, the next planned step is to extensively discuss this proposal with individuals with lived experience of post-COVID-19 condition to adapt the core outcome domains and appropriate instruments.

The recommendations here are meant for research and clinical application in adults. However, post-COVID-19 condition is also experienced by children and adolescents [155]. Although it is likely that most domains suggested here may also be essential to assess in younger individuals, there are important differences to assessments in adults, such as including the role and opinions of parents, the age-sensitive assessment of symptoms, and psychopathology, in the younger children; therefore, the optimal instruments may deviate from those suggested in this paper.

Finally, a recommended list as suggested here must always be considered a temporary spotlight that needs continuous evaluations, extensions, and adaptations.

### Future research

It is crucial to remember that all the variables and mechanisms mentioned previously are likely to interact with each other rather than functioning in isolation, which is the fundamental idea of the biopsychosocial model. However, it seems fruitful to identify those factors that are strong predictors for post-COVID-19 condition or are the mechanisms most amenable to change. To come to sound evidence-based and personalized conclusions regarding post-COVID-19 condition treatments, it is crucial to run clinical trials testing potential biomedical and multimodal psychosocial treatments and their effects on the domains we summarized under the higher-order dimension clinical aspects. A serious problem is that most studies lack an adequate control group to compare intervention effects or symptom prevalence between post-COVID-19 condition patients and individuals who fully recovered after the acute SARS-CoV-2 infection. In addition to that, linear models to explain patient burden seem to be insufficient, which is why complex, systemic models are necessary; this argues for network models and analyses that consider multiple variables and symptoms and their associations at the same time considering their complex interactions [156].

## Conclusions

Using and reporting identical core domains and agreed-upon outcomes within clinical studies on post-COVID-19 condition and using these recommended measures in clinical practice will speed up the accumulation of evidence-based knowledge regarding post-COVID-19 condition and the resulting best-possible treatment. Covering the psychosocial aspects of post-COVID-19 condition in the context of a broader biopsychosocial perspective seems to be a crucial factor in providing patients as well as healthcare professionals with the best healthcare possible. Psychosocial factors need to be considered as equally important as biomedical factors to develop a full understanding of illness trajectories and personalized intervention options. Personalized treatments require the identification of relevant subgroups, and our set of psychosocial domains could be a critical part of such a multidimensional phenotyping and classification procedure.

## Supplementary Information

Additional file 1.

Additional file 2.

## Data Availability

No datasets were generated or analysed during the current study.

## Abbreviations

ACE: Adverse childhood experiences
BDS: Bodily Distress Syndrome
BFI-10: Big Five Inventory-10
B-IPQ: Brief Illness Perception Questionnaire
CBRQ(-SF): Cognitive and Behavioral Responses to Symptoms Questionnaire (Short Version)
CCI: Charlson Comorbidity Index
CFS: Chalder Fatigue Scale
CoCo-P: Cognitive Complaints Questionnaire – Participation
COS: Core Outcome Set
CRP: C-reactive protein
DIPS: Diagnostic Interview for Mental Disorders
DSQ-PEM: DePaul Post-Exertional Malaise Questionnaire
EQ-5D-5L: European Quality of Life 5 Dimensions 5 Level Version
EURONET-SOMA: European Network to improve diagnostic, treatment and healthcare for patients with persistent somatic symptoms
GAD-7: Generalized Anxiety Disorder-7
G-EEE: Generic rating scale for previous treatment Experiences, treatment Expectations, and treatment Effects
ICU: Intensive care unit
IPAQ: International Physical Activity Questionnaire
ISI: Insomnia Severity Index
M.I.N.I.: Mini International Neuropsychiatric Review
MSPSS: Multidimensional Scale of Perceived Social Support
NRS: Numeric Rating Scale
OSSS-3: Oslo Social Support Scale
PANAS: Positive and Negative Affect Schedule
PCL-5: PTSD Checklist for DSM-5
PC-COS: Post-COVID Core Outcome Set
PHQ-4: Patient Health Questionnaire-4
PHQ-8: Patient Health Questionnaire-8
PHQ-9: Patient Health Questionnaire-9
PHQ-15: Patient Health Questionnaire-15
PRIME-MD: Primary Evaluation of Mental Disorders
PDI: Pain Disability Index
PROs: Patient-reported outcomes
PSQI: Pittsburgh Sleep Quality Index
PTSD: Post-traumatic stress disorder
QoL: Quality of Life
SCL-8: Symptom Checklist-8
SCAN: Schedules for Clinical Assessment in Neuropsychiatry
SCID-5: Structured Clinical Interview for DSM-5
SCQ: Self-administered Comorbidity Questionnaire
SF-12/SF-36: Short Form Health Survey
SSAS: Somatosensory Amplification Scale
SSS-8: Somatic Symptom Scale-8
SSD-12: Somatic Symptom Disorder – B criteria Scale
TAP: Test battery of Attentional Performance
TEX-Q: Treatment Expectation Questionnaire
UCLA: University of California, Los Angeles
ULS: UCLA Loneliness Scale
WHO: World Health Organization
WI-7: Whiteley Index-7
SARS-CoV-2: Severe Acute Respiratory Syndrome Coronavirus 2
