# Supplementary material for: Assessment of psychosocial aspects in adults in post-COVID-19 condition: the EURONET-SOMA recommendations on core outcome domains for clinical and research use
Source: BMC Med. 2025 Feb 11;23:81. doi: 10.1186/s12916-025-03927-0 (PMC11818037; doi:10.1186/s12916-025-03927-0)
Supplement: Supplementary file 1 — Additional file 1. [file 12916_2025_3927_MOESM1_ESM.docx]

**Additional File 1: Table S1**

Table S1. Higher-order dimensions, domains, subdomains with specifications and examples of instruments to cover the psychosocial aspects of post-COVID-19 condition in a biopsychosocial model (Instruments with * are part of the Clinical Core Set, Table 1).

| **Domain** | **Subdomain** | **Specifications** | **Instruments/examples** |
| --- | --- | --- | --- |
| **I. Outcomes** | | |  |
| Classification/ Diagnostic of post-COVID-19 condition | SARS-CoV-2 infection | - Time point of SARS-CoV-2 infection/COVID-19 diagnosis - PCR vs. self-report only - Onset and duration of original COVID-19 symptoms/post-COVID-19 condition symptoms - Make a clear distinction between onset of novel symptoms vs. reoccurrence of previous symptoms and/or exacerbation of pre-existent symptoms - Treatment of COVID-19 (at home, hospital, normal ward/ICU, length of stay at hospital) - Number of infections | - Single items - SARS-CoV-2 IgG antibody tests |
|  | Vaccination status | - Number of vaccinations - Time point of vaccination - Type of vaccine (e.g., Biontech, Moderna, Astrazeneca, …) |  |
| Somatic symptoms (incl. fatigue); of note, assessing the symptom duration for all complaints in this section is recommended | Fatigue | - Fatigue and Post-Exertional Malaise (PEM) | - Post Covid Functional Status (PCFS) (1) - Chalder Fatigue Scale (2) - DePaul Symptom Questionnaire–Post-Exertional Malaise (DSQ-PEM) (3) |
|  | Other somatic symptoms | - Somatic symptom burden/severity | - Patient Health Questionnaire-15 (PHQ-15) (4) - Screening for Somatoform Symptoms (SOMS-7) (5) - ***Somatic Symptom Scale (SSS-8)** (6) - Bodily Distress Syndrome (BDS) Checklist (7) - Numeric rating scale (NRS) (0..10) |
|  |  | - Symptom Intensity | - COVID‐19 Yorkshire Rehabilitation Scale (C19‐YRS) (8) - Somatic symptom intensity EURONET-SOMA Numeric Rating Scale (9) |
|  |  | - Pain | - Numeric rating scale (NRS), see ICD-11 Chronic Pain section (0..10) (10) |
|  |  | - Shortness of breath | - Dyspnoea-12 (11) |
|  |  | - Impairment of smell and taste | Sinonasal outcome test (SNOT-22)(12) |
|  |  | - Hyperventilation syndrome | Nijmegen questionnaire (13) |
|  |  | - Sleep | - Pittsburgh Sleep Quality Inventory (PSQI) (14) - Insomnia Severity Index(ISI) (15) |
| Psychopathological status and mental comorbidities | Psychopathological status | - (Health) Anxiety | - Generalized Anxiety Disorder-7 (GAD-7) (16) - ***Whiteley Index (WI-7)** (17), revised 6-item version (18) |
|  |  | - Depression | Patient Health Questionnaire-9 (PHQ-9) (16), 8-item-version (PHQ-8) (19), ***PHQ-4 (2 items for depression, 2 items for anxiety)** (20)  Symptom Checklist-8 (SCL-8) with four items for anxiety (SCL-4anx subscale) and four items for depressive symptoms (SCL-4dep) (21)   - Hospital Anxiety and Depression Scale (HADS) (22) |
|  |  | - Post-traumatic stress disorder (PTSD) | - PTSD Checklist for DSM-5 (PCL-5) (23) (asking regarding COVID-19 related traumatic events) |
|  | Mental comorbidities | - Validated structured clinical interview - Preexisting conditions (history/time scale of mental disorders) - Duration/onset of symptoms. Pre-treatments. | - Structured Clinical Interview for DSM-5 (SCID-5) (24) - Diagnostic Interview for mental disorders (DIPS) (53) - Primary Care Evaluation of Mental Disorders (PRIME-MD) (25) - Mini-International Neuropsychiatric Review   (M.I.N.I.) (26) |
| Physical status and somatic comorbidity | Somatic comorbidity | - Preexisting medical conditions - Duration/onset of symptoms - Pre-treatments - Current medications - Persistent symptoms in the past and their attribution to an infection (Lyme borreliosis, etc.) or to environmental factors (idiopathic environmental intolerance, etc.). | - Structured medical history, hospital charts, use ICD-10/11 - Charlson Comorbidity index (CCI) (27) - Self-Administered Comorbidity Questionnaire (SCQ)(28) |
|  | Physical status | - Functional status | - 6 minute walk test (29), - one minute sit to stand test (30) - JAMAR® grip strength test (31) |
| Neurocognitive Symptoms | Subjective | - Subjective cognitive complaints | - Cognitive complaints – participation (CoCoP) (32) - Subjective report of difficulties experienced by patients in daily life: Behavioral Rating Inventory of Executive Function (BRIEF) |
|  | Objective | - Attention, (working) Memory, long-term memory, executive functions | - Test of Attentional Performance (TAP) (33) - Montreal Cognitive Assessment Test (MoCa) |
| Illness consequences | Disability | - Illness-related disability | - ***Adapted Pain Disability Index (PDI)** (34) - World Health Organization Disability Assessment Schedule 2.0 (WHODAS 2.0) (35) |
|  | Quality of life |  | - 12-items Short Form Health Survey (SF-12) (36) - European Quality of Life 5 Dimensions 5 Level Version (EQ-5D-5L) (37) |
|  | Health care use | - Number of consultations and different medical specialists - medical exams - physical therapy - speech therapy - occupational therapy - nutrition therapy | - Schedules for Clinical Assessment in Neuropsychiatry (SCAN) (38) - Single items (39) |
|  | Work | - Working ability - Occupational status during the pandemic: Health worker, remote work. - Occupational consequences of COVID-19: loss of employment, loss of incomes - Satisfaction at work | - Single question |
| **II. Mechanisms (present in ill and healthy individuals)** | | |  |
| Cognitive components | Illness perceptions/beliefs | - Beliefs about the causes of symptoms | - (Brief) Illness Perception Questionnaire (BIPQ) (40) |
|  | Cognitive appraisal processes | - Perceived stress | - Perceived stress scale (PSS-10) (41) |
|  |  | - Catastrophizing and fear avoidance | - ***Somatic Symptom Disorder – B Criteria Scale (SSD-12)**(42) - Cognitive and Behavioural Responses to Symptoms Questionnaire (CBRQ) (43) |
|  |  | - Somatosensory amplification | - Somatosensory Amplification Scale (SSAS) (44) |
|  |  | - Interoception | - Heart perception/ Schandry task (45) |
|  |  | - Environmental worries | - Modern health worries scale (MHWS) (46) |
|  | Expectations | - Expectations of symptom severity, symptom burden, duration/course of symptoms, coping with symptoms and treatment effects | - ***Generic Rating for Treatment pre-Experiences, Treatment Expectations, and Treatment Effects (G-EEE)** (47) - Treatment Expectation Questionnaire (TEX-Q) (48) |
|  |  | - Treatment effects | - Generic Rating for Treatment pre-Experiences, Treatment Expectations, and Treatment Effects (G-EEE) (47) - Treatment Expectation Questionnaire (TEX-Q) (48) |
|  |  | - treatment satisfaction/ recommendation item | - Single item numeric rating scale |
|  |  | - adverse treatment effects | - Generic Rating for Treatment pre-Experiences, Treatment Expectations, and Treatment Effects (G-EEE) (47) - General Assessment of Side Effects (GASE) (49) |
| Affective components | Negative affectivity | - Negative affect | - Somatic Symptom Disorder – B Criteria Scale (SSD-12)(42) - Positive and Negative Affectivity Schedule (PANAS) (50), 10-item short form (51) |
|  |  | - Coping/Emotional regulation | - Emotion Regulation Questionnaire (ERQ) |
| Behavioral components | Illness behavior |  | - Somatic Symptom Disorder – B Criteria Scale (SSD-12)(42) |
|  | Activity level | - Physical activity | - International Physical Activity Questionnaire (IPAQ) (52) - Wearable Activity tracker |
|  | Fear avoidance | - Avoidance behavior | - Fear-Avoidance Beliefs Questionnaire (FABQ) |
| Social components |  | - Social support | - The Multidimensional Scale of Perceived Social Support (MSPSS) (53) - Oslo Social Support Scale (OSSS-3) (54) |
|  |  | - Perceived stigmatization | - Single Items |
|  |  | - Conditions of lockdown in the living area (loneliness, etc.) |  |
|  |  | - COVID-19 cases in relatives or friends |  |
|  |  | - Relation with associations of patients |  |
| Psychobiological bridge markers |  | - (Neuro-)Inflammation | - High-sensitivity CRP (in serum/plasma and cerebrospinal fluid) - Pro-inflammatory cytokines (IL-6, IL-8, TNF-α) |
|  |  | - Cardiac dysfunction | - NTproBNP |
|  |  | - Neuroendocrinological markers | - Hair or saliva 9 |
|  |  | - Vitamins |  |
|  |  | - Autonomic dysfunction | - Heart rate variability (HRV) |
|  |  | - Coagulation / thrombotic tendency | - D-dimer |
| **III. Risk factors** | | | |
| Sociodemographic status and sociocultural factors |  | - Age, sex, gender, race | Single items |
|  |  | - Education | Single item |
|  |  | - Socioeconomic status | Single item |
|  |  | - Loneliness | UCLA Loneliness Scale (ULS) (55), single item (56) |
| Pre-existing mental or somatic health issues |  |  | - Single questions (e.g., about pretreatments) - Please see recommendations in the domains: Psychopathological status and mental comorbidities - Physical status and somatic comorbidity |
| Personality Traits (neuroticism) |  |  | - Big Five Inventory-10 (BFI-10) (57) |
| Adverse childhood experiences |  |  | - Adverse Childhood Experiences Questionnaire (ACE) (58) |
|  |  |  | - Question about psychological trauma before COVID |
| Ongoing disability or pension claim |  |  | - Single question |
| Social media use |  |  | - Single question |
|  |  | - Social environment (family or friends with post-COVID-19) | - Single question |

**References**

1. Klok FA, Boon GJAM, Barco S, Endres M, Miranda Geelhoed JJ, Knauss S, u. a. The post-COVID-19 functional status scale: A tool to measure functional status over time after COVID-19. European Respiratory Journal. 2020;56(1):10–2.

2. Chalder T, Berelowitz G, Pawlikowska T, Watts L, Wessely S, Wright D, u. a. Development of a fatigue scale. Journal of psychosomatic research. 1993;37(2):147–53.

3. Cotler J, Holtzman C, Dudun C, Jason L. A Brief Questionnaire to Assess Post-Exertional Malaise. Diagnostics. 11. September 2018;8(3):66.

4. Kroenke K, Spitzer RL, Williams JBW. The PHQ-15: Validity of a New Measure for Evaluating the Severity of Somatic Symptoms: Psychosomatic Medicine. März 2002;64(2):258–66.

5. Kleinstäuber M, Rief W, Hiller W. the Screening of Somatoform Disorders (Soms): Development of a New Distress Index. Verhaltenstherapie & Verhaltensmedizin. 2013;34(4):361–77.

6. Gierk B, Kohlmann S, Kroenke K, Spangenberg L, Zenger M, Brähler E, u. a. The Somatic Symptom Scale–8 (SSS-8): A Brief Measure of Somatic Symptom Burden. JAMA Intern Med. 1. März 2014;174(3):399.

7. Petersen MW, Rosendal M, Ørnbøl E, Fink P, Jørgensen T, Dantoft TM, u. a. The BDS checklist as measure of illness severity: a cross-sectional cohort study in the Danish general population, primary care and specialised setting. BMJ Open. Dezember 2020;10(12):e042880.

8. O’Connor RJ, Preston N, Parkin A, Makower S, Ross D, Gee J, u. a. The COVID‐19 Yorkshire Rehabilitation Scale (C19‐YRS): Application and psychometric analysis in a post‐COVID‐19 syndrome cohort. Journal of Medical Virology. März 2022;94(3):1027–34.

9. Rief W, Burton C, Frostholm L, Henningsen P, Kleinstäuber M, Kop WJ, u. a. Core Outcome Domains for Clinical Trials on Somatic Symptom Disorder, Bodily Distress Disorder, and Functional Somatic Syndromes: European Network on Somatic Symptom Disorders Recommendations. Psychosom Med. November 2017;79(9):1008–15.

10. Hay G, Korwisi B, Rief W, Smith BH, Treede RD, Barke A. Pain severity ratings in the 11th revision of the International Classification of Diseases: a versatile tool for rapid assessment. Pain. Dezember 2022;163(12):2421–9.

11. Yorke J, Moosavi SH, Shuldham C, Jones PW. Quantification of dyspnoea using descriptors: development and initial testing of the Dyspnoea-12. Thorax. 1. Januar 2010;65(1):21–6.

12. Kennedy JL, Hubbard MA, Huyett P, Patrie JT, Borish L, Payne SC. Sino-nasal outcome test (SNOT-22): A predictor of postsurgical improvement in patients with chronic sinusitis. Annals of Allergy, Asthma & Immunology. Oktober 2013;111(4):246-251.e2.

13. van Dixhoorn J, Folgering H. The Nijmegen Questionnaire and dysfunctional breathing. ERJ Open Res. Mai 2015;1(1):00001–2015.

14. Buysse DJ, Reynolds CF, Monk TH, Berman SR, Kupfer DJ. The Pittsburgh sleep quality index: A new instrument for psychiatric practice and research. Psychiatry Research. Mai 1989;28(2):193–213.

15. Morin CM, Belleville G, Bélanger L, Ivers H. The Insomnia Severity Index: Psychometric Indicators to Detect Insomnia Cases and Evaluate Treatment Response. Sleep. Mai 2011;34(5):601–8.

16. Kroenke K, Spitzer RL, Williams JBW, Löwe B. The Patient Health Questionnaire Somatic, Anxiety, and Depressive Symptom Scales: a systematic review. General Hospital Psychiatry. Juli 2010;32(4):345–59.

17. Conradt M, Cavanagh M, Franklin J, Rief W. Dimensionality of the Whiteley Index: Assessment of hypochondriasis in an Australian sample of primary care patients. Journal of Psychosomatic Research. Februar 2006;60(2):137–43.

18. Carstensen TBW, Ørnbøl E, Fink P, Pedersen MM, Jørgensen T, Dantoft TM, u. a. Detection of illness worry in the general population: A specific item on illness rumination improves the Whiteley Index. Journal of Psychosomatic Research. November 2020;138:110245.

19. Kroenke K, Strine TW, Spitzer RL, Williams JBW, Berry JT, Mokdad AH. The PHQ-8 as a measure of current depression in the general population. Journal of Affective Disorders. April 2009;114(1–3):163–73.

20. Kroenke K, Spitzer RL, Williams JBW, Lowe B. An Ultra-Brief Screening Scale for Anxiety and Depression: The PHQ-4. Psychosomatics. 1. November 2009;50(6):613–21.

21. Fink P, Jensen J, Borgquist L, Brevik JI, Dalgard OS, Sandager I, u. a. Psychiatric morbidity in primary public health care: a Nordic multicentre investigation. Part I: method and prevalence of psychiatric morbidity. Acta Psychiatr Scand. Dezember 1995;92(6):409–18.

22. Zigmond AS, Snaith RP. The hospital anxiety and depression scale. Acta psychiatrica Scandinavica. Juni 1983;67(6):361–70.

23. Blevins CA, Weathers FW, Davis MT, Witte TK, Domino JL. The Posttraumatic Stress Disorder Checklist for *DSM-5* (PCL-5): Development and Initial Psychometric Evaluation: Posttraumatic Stress Disorder Checklist for *DSM-5*. JOURNAL OF TRAUMATIC STRESS. Dezember 2015;28(6):489–98.

24. First MB, Williams JBW, Karg RS, Spitzer RL. SCID-5-CV: Structured Clinical Interview for DSM-5 Disorders: clinician version. Arlington, VA: American Psychiatric Association Publishing; 2016. 95 S.

25. Spitzer RL, Williams JB, Kroenke K, Linzer M, deGruy FV, Hahn SR, u. a. Utility of a new procedure for diagnosing mental disorders in primary care. The PRIME-MD 1000 study. JAMA. 14. Dezember 1994;272(22):1749–56.

26. Sheehan DV. The Mini-International Neuropsychiatric Interview (M.I.N.I.): The Development and Validation of a Structured Diagnostic Psychiatric Interview for DSM-IV and ICD-10. J Clin Psychiatry.

27. Charlson ME, Pompei P, Ales KL, MacKenzie CR. A new method of classifying prognostic comorbidity in longitudinal studies: Development and validation. Journal of Chronic Diseases. Januar 1987;40(5):373–83.

28. Sangha O, Stucki G, Liang MH, Fossel AH, Katz JN. The self-administered comorbidity questionnaire: A new method to assess comorbidity for clinical and health services research. Arthritis & Rheumatism. 15. April 2003;49(2):156–63.

29. Enright PL. The Six-Minute Walk Test. RESPIRATORY CARE. 2003;48(8).

30. Spence JG, Brincks J, Løkke A, Neustrup L, Østergaard EB. One-minute sit-to-stand test as a quick functional test for people with COPD in general practice. npj Prim Care Respir Med. 15. März 2023;33(1):11.

31. Leong DP, Teo KK, Rangarajan S, Lopez-Jaramillo P, Avezum A, Orlandini A, u. a. Prognostic value of grip strength: findings from the Prospective Urban Rural Epidemiology (PURE) study. The Lancet. Juli 2015;386(9990):266–73.

32. Spreij LA, Sluiter D, Gosselt IK, Visser-Meily JMA, Nijboer TCW. CoCo - participation: The development and clinical use of a novel inventory measuring cognitive complaints in daily life. Neuropsychological Rehabilitation. 7. Februar 2021;31(2):255–77.

33. Zimmermann P, Fimm B. Testbatterie zur Aufmerksamkeitsprüfung. Würselen: Psytest; 1994.

34. Mewes R, Rief W, Stenzel N, Glaesmer H, Martin A, Brähler E. What is „normal“ disability? An investigation of disability in the general population. Pain. März 2009;142(1–2):36–41.

35. Üstün TB, Chatterji S, Kostanjsek N, Rehm J, Kennedy C, Epping-Jordan J, u. a. Developing the World Health Organization Disability Assessment Schedule 2.0. Bull World Health Organ. 1. November 2010;88(11):815–23.

36. Ware J, Kosinski M, Keller SD. A 12-Item Short-Form Health Survey: construction of scales and preliminary tests of reliability and validity. Medical care. März 1996;34(3):220–33.

37. Herdman M, Gudex C, Lloyd A, Janssen Mf, Kind P, Parkin D, u. a. Development and preliminary testing of the new five-level version of EQ-5D (EQ-5D-5L). Qual Life Res. Dezember 2011;20(10):1727–36.

38. Schedules for Clinical Assessment in Neuropsychiatry Sample for personal inspection only WHO Schedules for Clinical Assessment in Neuropsychiatry version 2.1 2 CONTENTS. In. Verfügbar unter: http://whoscan.org/wp-content/uploads/2014/10/xinterview.pdf

39. Löwe B, Andresen V, Van den Bergh O, Huber TB, von dem Knesebeck O, Lohse AW, u. a. Persistent SOMAtic symptoms ACROSS diseases — from risk factors to modification: scientific framework and overarching protocol of the interdisciplinary SOMACROSS research unit (RU 5211). BMJ Open. Januar 2022;12(1):e057596.

40. Broadbent E, Petrie KJ, Main J, Weinman J. The brief illness perception questionnaire. Journal of psychosomatic research. Juni 2006;60(6):631–7.

41. Cohen S, Kamarck T, Mermelstein R. A global measure of perceived stress. J Health Soc Behav. Dezember 1983;24(4):385–96.

42. Toussaint A, Löwe B, Brähler E, Jordan P. The Somatic Symptom Disorder - B Criteria Scale (SSD-12): Factorial structure, validity and population-based norms. Journal of Psychosomatic Research. Juni 2017;97:9–17.

43. Picariello F, Chilcot J, Chalder T, Herdman D, Moss‐Morris R. The Cognitive and Behavioural Responses to Symptoms Questionnaire (CBRQ): Development, reliability and validity across several long‐term conditions. British J Health Psychol. Mai 2023;28(2):619–38.

44. Barsky AJ, Wyshak G. Hypochondriasis and Somatosensory Amplification. Br J Psychiatry. September 1990;157(3):404–9.

45. Schandry. Heart Beat Perception and Emotional Experience.pdf.

46. Petrie KJ, Sivertsen B, Hysing M, Broadbent E, Moss-Morris R, Eriksen HR, u. a. Thoroughly modern worries The relationship of worries about modernity to reported symptoms, health and medical care utilization$. Journal of Psychosomatic Research. 2001;7.

47. Rief W, Nestoriuc Y, Mueller EM, Hermann C, Schmidt K, Bingel U. Generic rating scale for previous treatment experiences , treatment expectations , and treatment effects ( GEEE ) Generic rating scale for previous treatment experiences , treatment expectations , and treatment effects ( GEEE ) University of Duisburg-Esse. 2021;(April).

48. Alberts J, Löwe B, Glahn MA, Petrie K, Laferton J, Nestoriuc Y, u. a. Development of the generic, multidimensional Treatment Expectation Questionnaire (TEX-Q) through systematic literature review, expert surveys and qualitative interviews. BMJ Open. 2020;10(8).

49. Rief W, Barsky AJ, Glombiewski JA, Nestoriuc Y, Glaesmer H, Braehler E. Assessing general side effects in clinical trials: Reference data from the general population. Pharmacoepidemiology and Drug Safety. 2011;20(4):405–15.

50. Watson D, Clark LA, Tellegen A. Development and validation of brief measures of positive and negative affect: The PANAS scales. Journal of Personality and Social Psychology. 1988;54(6):1063–70.

51. Thompson ER. Development and Validation of an Internationally Reliable Short-Form of the Positive and Negative Affect Schedule (PANAS). Journal of Cross-Cultural Psychology. März 2007;38(2):227–42.

52. Craig CL, Marshall AL, Sjöström M, Bauman AE, Booth ML, Ainsworth BE, u. a. International physical activity questionnaire: 12-country reliability and validity. Medicine and science in sports and exercise. August 2003;35(8):1381–95.

53. Zimet GD, Dahlem NW, Zimet SG, Farley GK. The Multidimensional Scale of Perceived Social Support. Journal of Personality Assessment. März 1988;52(1):30–41.

54. Kocalevent RD, Berg L, Beutel ME, Hinz A, Zenger M, Härter M, u. a. Social support in the general population: standardization of the Oslo social support scale (OSSS-3). BMC Psychol. Dezember 2018;6(1):31.

55. Russell DW. UCLA Loneliness Scale (Version 3): Reliability, Validity, and Factor Structure. Journal of Personality Assessment. Februar 1996;66(1):20–40.

56. Shankar A, McMunn A, Banks J, Steptoe A. Loneliness, social isolation, and behavioral and biological health indicators in older adults. Health Psychology. 2011;30(4):377–85.

57. Rammstedt B, John OP. Measuring personality in one minute or less: A 10-item short version of the Big Five Inventory in English and German. Journal of Research in Personality. Februar 2007;41(1):203–12.

58. Felitti VJ, Anda RF, Nordenberg D, Williamson DF, Spitz AM, Edwards V, u. a. Relationship of childhood abuse and household dysfunction to many of the leading causes of death in adults: The adverse childhood experiences (ACE) study. American Journal of Preventive Medicine. 1998;14(4):245–58.
