# Supplementary material for: Assessment of psychosocial aspects in adults in post-COVID-19 condition: the EURONET-SOMA recommendations on core outcome domains for clinical and research use
Source: BMC Med. 2025 Feb 11;23:81. doi: 10.1186/s12916-025-03927-0 (PMC11818037; doi:10.1186/s12916-025-03927-0)
Supplement: Supplementary file 2 — Additional file 2. [file 12916_2025_3927_MOESM2_ESM.pdf]

Somatic Symptom Scale – 8 (SSS-8)

**During the past 7 days, how much have you been bothered by any of the following problems?**					
	Not at all	A little bit	Somewhat	Quite a bit	Very much
Stomach or bowel problems	0	1	2	3	4
Back pain	0	1	2	3	4
Pain in your arms, legs, or joints	0	1	2	3	4
Headaches	0	1	2	3	4
Chest pain or shortness of breath	0	1	2	3	4
Dizziness	0	1	2	3	4
Feeling tired or having low engery	0	1	2	3	4
Trouble sleeping	0	1	2	3	4

Gierk B, Kohlmann S, Kroenke K, Spangenberg L, Zenger M, Brähler E, & Löwe B. (2014). The Somatic Symptom Scale–8 (SSS-8): A brief measure of somatic symptom burden. JAMA Internal Medicine, 174(3), 399–407

PHQ-4

Over the last 2 weeks, how often have you been bothered by the following problems?	Not at all	Several days	More than half the days	Nearly every day
1. Feeling nervous, anxious or on edge	0	1	2	3
2. Not being able to stop or control worrying	0	1	2	3
3. Little interest or pleasure in doing things	0	1	2	3
4. Feeling down, depressed, or hopeless	0	1	2	3

Kroenke K, Spitzer RL, Williams JB, Löwe B. An ultra-brief screening scale for anxiety and depression: the PHQ-4. Psychosomatics. 2009 Nov-Dec;50(6):613-21. doi: 10.1176/appi.psy.50.6.613

Whiteley Index-7

Within the past 12 months, to what extent have you been bothered by:

		**'Not at all'**	**'A bit'**	**'Somewhat'**	**'Quite a lot'**	**'A lot'**
		0	1	2	3	4
1.	Worries that there is something seriously wrong with your body?					
2.	Worries that you suffer from a disease you have read or heard about?					
3.	Worries about the possibility of having a serious illness?					
4.	Many different symptoms?					
5.	Thoughts, that the doctor may be wrong if telling you not to worry?					
6.	Worries about your health?					
7.	Recurrent thoughts about being ill that are difficult to get off your mind?					

Generic rating scale for previous treatment experiences, treatment expectations, and treatment effects (G-EEE)

English version:

Expectations

The following section refers to what **expectations** you have for the treatment:												
**1. How much improvement** ([insert: primary outcome]) **do you expect from the treatment** (optional: add name of treatment)**?**												
no improvement	☐ 0	☐ 1	☐ 2	☐ 3	☐ 4	☐ 5	☐ 6	☐ 7	☐ 8	☐ 9	☐ 10	greatest improvement imaginable
**2. How much worsening of** ([insert: primary outcome]) **do you expect from the treatment** (optional: add name of treatment)**?**												
no worsening	☐ 0	☐ 1	☐ 2	☐ 3	☐ 4	☐ 5	☐ 6	☐ 7	☐ 8	☐ 9	☐ 10	greatest worsening imaginable
3. **How many complaints/side effects do you expect from the treatment** (optional: add name of treatment)**?**												
no complaints	☐ 0	☐ 1	☐ 2	☐ 3	☐ 4	☐ 5	☐ 6	☐ 7	☐ 8	☐ 9	☐ 10	greatest complaints imaginable

Previous treatment experiences

4. The following section is about **previous treatment experiences** you had with (specify treatment):												
**I have never experienced this treatment.** *(continue with question 8)*												☐
**I have experienced this treatment during the last 12 months (nearly) daily.**												☐
**I have experienced this treatment during the last 12 months on more than 10 days.**												☐
**I have experienced this treatment during the last 12 months on about 5 to 10 days.**												☐
**I have experienced this treatment during the last 12 months on about 1 to 4 days.**												☐
**I have not experienced this treatment during the last 12 months, but I have experienced it before.**												☐
**5. How much improvement of** ([insert: primary outcome]) **have you experienced with the treatment** (optional: add name of treatment) **in the past?**												
no improvement	☐ 0	☐ 1	☐ 2	☐ 3	☐ 4	☐ 5	☐ 6	☐ 7	☐ 8	☐ 9	☐ 10	greatest improvement imaginable

**6. How much worsening of** ([insert: primary outcome]) **have you experienced with the treatment** (optional: add name of treatment) **in the past?**												
no worsening	☐ 0	☐ 1	☐ 2	☐ 3	☐ 4	☐ 5	☐ 6	☐ 7	☐ 8	☐ 9	☐ 10	greatest worsening imaginable
**7. How many complaints/side effects have you experienced with the treatment** (optional: add name of treatment) **in the past?**												
no complaints	☐ 0	☐ 1	☐ 2	☐ 3	☐ 4	☐ 5	☐ 6	☐ 7	☐ 8	☐ 9	☐ 10	greatest complaints imaginable

Current treatment effects

The following section is about what **changes you have experienced since your participation in this study**:												
**8. How much improvement of** ([insert: primary outcome]) **have you experienced since?**												
no improvement	☐ 0	☐ 1	☐ 2	☐ 3	☐ 4	☐ 5	☐ 6	☐ 7	☐ 8	☐ 9	☐ 10	greatest improvement imaginable
**9. How much worsening of** ([insert: primary outcome]) **have you experienced since?**												
no worsening	☐ 0	☐ 1	☐ 2	☐ 3	☐ 4	☐ 5	☐ 6	☐ 7	☐ 8	☐ 9	☐ 10	Greatest worsening imaginable
**10. How many complaints/side effects have you experienced** since your participation/treatment with (insert: name of treatment)**?**												
no complaints	☐ 0	☐ 1	☐ 2	☐ 3	☐ 4	☐ 5	☐ 6	☐ 7	☐ 8	☐ 9	☐ 10	greatest complaints imaginable

Completion of study (this is an optional question if you want to evaluate the assumptions of the patient).

Thank you for your participation. In this study, you received a specific treatment (pill, cream, or similar). Regardless of the information provided by the study investigator, what do you believe regarding your treatment?

I believe this treatment (pill, cream) contained a real active ingredient.

☐

I believe this treatment (pill, cream) did not contain a real active ingredient.

☐

Rief, W., Nestoriuc, Y., Mueller, E. M., Hermann, C., Schmidt, K., & Bingel, U. (2021). Generic rating scale for previous treatment experiences, treatment expectations, and treatment effects (GEEE). PsychArchives. https://doi.org/10.23668/PSYCHARCHIVES.4717

SSD-12

	Never	Rarely	Sometimes	Often	Very often
1. I think that my physical symptoms are signs of a serious illness (I).	□	□	□	□	□
2. I am very worried about my health (II).	□	□	□	□	□
3. My health concerns hinder me in everyday life (III).	□	□	□	□	□
4. I am convinced that my symptoms are serious (I).	□	□	□	□	□
5. My symptoms scare me (II).	□	□	□	□	□
6. My physical complaints occupy me for most of the day (III).	□	□	□	□	□
7. Others tell me that my physical problems are not serious (I).	□	□	□	□	□
8. I'm worried that my physical complaints will never stop (II).	□	□	□	□	□
9. My worries about my health take my energy (III).	□	□	□	□	□
10. I think that doctors do not take my physical complaints seriously (I).	□	□	□	□	□
11. I am worried that my physical symptoms will continue into the future (II).	□	□	□	□	□
12. Due to my physical complaints, I have poor concentration on other things (III).	□	□	□	□	□

Toussaint A, Löwe B, Brähler E, Jordan P. The Somatic Symptom Disorder - B Criteria Scale (SSD-12): Factorial structure, validity and population-based norms. J Psychosom Res. 2017 Jun;97:9-17. doi: 10.1016/j.jpsychores.2017.03.017.

Adapted version of the Pain Disability Index (PDI)

Instruction: For each of the following aspects, please select the point on the scale that you feel is most appropriate in describing you on a scale from None (0) to Total (10).

1 Family and home responsibilities

Activities related to home and family

0	1	2	3	4	5	6	7	8	9	10

No Disability — Worst Disability

2 Recreation

Hobbies sports and other leisure time activities

0	1	2	3	4	5	6	7	8	9	10

No Disability — Worst Disability

3 Social activity

Participation with friends and acquaintances other than family members

0	1	2	3	4	5	6	7	8	9	10

No Disability — Worst Disability

4 Occupation

Activities partly or directly related to working including housework or volunteering

0	1	2	3	4	5	6	7	8	9	10

No Disability — Worst Disability

5 Sexual behavior

Frequency and quality of sex life

0	1	2	3	4	5	6	7	8	9	10

No Disability — Worst Disability

6 Self care

Personal maintenance and independent daily living (bathing dressing etc.)

0	1	2	3	4	5	6	7	8	9	10

No Disability — Worst Disability

7 Life-support activity

Basic life-supporting behaviors (eating sleeping breathing etc.)

0	1	2	3	4	5	6	7	8	9	10

No Disability — Worst Disability

Mewes R, Rief W, Stenzel N, Glaesmer H, Martin A, Brähler E. What is "normal" disability? An investigation of disability in the general population. Pain. 2009 Mar;142(1-2):36-41. doi: 10.1016/j.pain.2008.11.007.
